# Cutaneous mucinosis of infancy: a rare case of joint involvement

**DOI:** 10.1186/s12969-021-00590-6

**Published:** 2021-06-29

**Authors:** Cristina Morreale, Dario Bleidl, Angela Rita Sementa, Clara Malattia

**Affiliations:** 1 Department of “Clinica Pediatrica e Reumatologia”, IRCCS Giannina Gaslini, Genoa, Italy; 2 Dipartimento di Neuroscienze, Riabilitazione, Oftalmologia, Genetica e Scienze Materno-Infantili, Università degli Studi di Genova, Genoa, Italy; 3 Department of “Dermatologia e Centro Angiomi”, IRCCS Giannina Gaslini, Genoa, Italy; 4 Department of “Anatomia Patologica”, IRCCS Giannina Gaslini, Genoa, Italy

**Keywords:** Cutaneous mucinosis, Arthritis, Baker’s cyst

## Abstract

**Background:**

Primary cutaneous mucinosis are a heterogeneous group of diseases characterized by the deposition of glycosaminoglycans in the dermis and the follicles. These diseases are rare in children therefore their diagnosis and management are still challenging. Joint involvement has been reported in patients with secondary cutaneous mucinosis and, rarely, in primary mucinosis. We describe a case of Cutaneous Mucinosis of Infancy with joint involvement.

**Case presentation:**

An healthy 5-year-old boy showed acute arthritis of the left knee and left elbow confirmed by ultrasound. Laboratory tests were within normal range. Symptoms disappeared after a course of nonsteroid anti-inflammatory drugs. One year later, the knee swelling reappeared; juvenile idiopathic arthritis was diagnosed and intra-articular steroid injection was performed. Due to persistence of arthritis of the knee he was admitted to our hospital. On physical examination variable skin-colored lesions were observed, which had been in existence for over 2 years.

We performed a skin biopsy that showed an interstitial mucine deposition in the reticular dermis. Cutaneous Mucinosis of Infancy was diagnosed.

**Discussion and conclusions:**

Cutaneous Mucinosis of Infancy is a persistent dermatosis with benign prognosis and no treatment is generally required. Our case report is particularly interesting because it is the first in which joint involvement has been reported in CMI, a disorder that has so far been described as limited to skin involvement. Further studies will be necessary in order to clarify the pathogenesis of joint involvement in primary mucinosis.

## Background

Cutaneous mucinosis covers a group of rare infiltrative diseases of unknown etiology that are characterized by the deposition of mucinous material in the dermis. Mucin is essentially composed of glycosaminoglycans (GAGs), which are long, polyanionic, polysaccharide polymers consisting of repeating disaccharide units. Cutaneous mucinosis are divided into two groups: primary forms, in which the mucin deposit is a distinctive histopathological feature, and secondary forms in which mucin deposition is associated with systemic diseases, including rheumatic disorders, such as systemic lupus erythematosus and dermatomyositis [1]. The classification of primary cutaneous mucinosis occurring in childhood is difficult and confusing because of the limited number of described cases, overlaps in their clinicopathological features and the lack of reports of homogeneous case series [2]. Primary cutaneous mucinosis in pediatrics includes the following rare disorders: Cutaneous Mucinosis of Infancy (CMI), Self-Healing Juvenile Cutaneous Mucinosis (SHJCM), follicular mucinosis, acral persistent papular mucinosis, hereditary progressive mucinous histiocytosis. Joint involvement has been described in some patients with secondary cutaneous mucinosis associated to systemic rheumatic diseases and, rarely, in primary mucinosis [2, 3]. We report a case of CMI with joint involvement.

## Case presentation

**Fig. 1:**
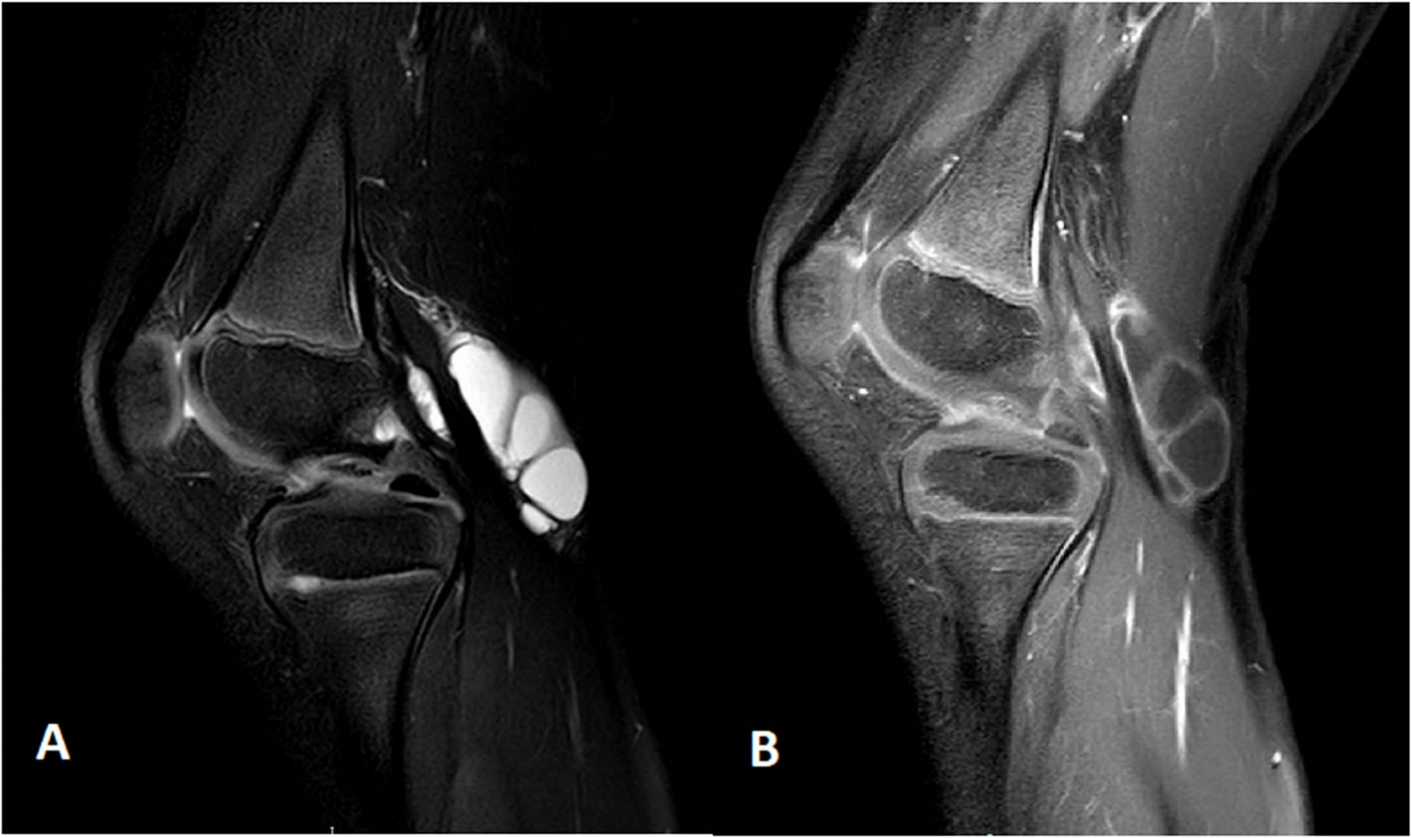
Magnetic Resonance Imaging (MRI). MRI sagittal view of the left knee: T2-weighted image (**A**), and T1-weighted contrast-enhanced image (**B**) showing the presence of a voluminous multiloculated Baker’s cyst

**Fig. 2:**
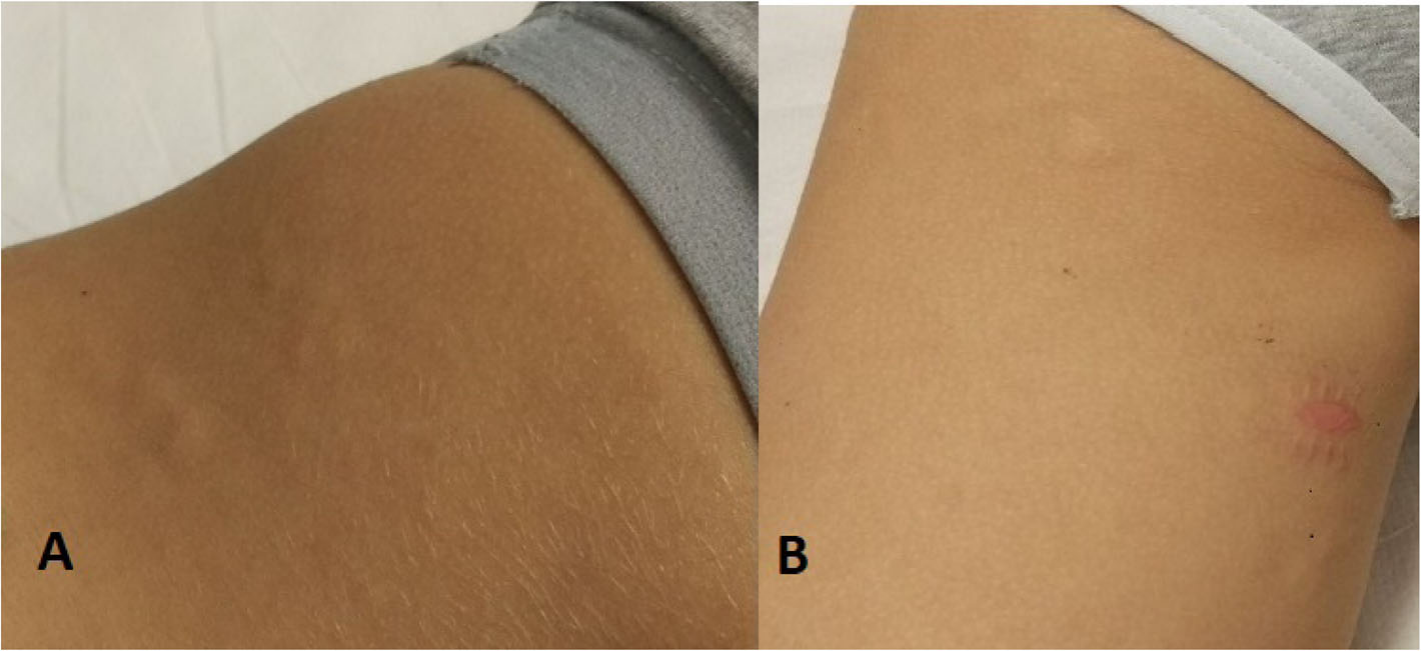
Skin lesions. **A** nodular lesions and plaques in the lumbar region. **B** nodular lesions in the femoral region

**Fig. 3:**
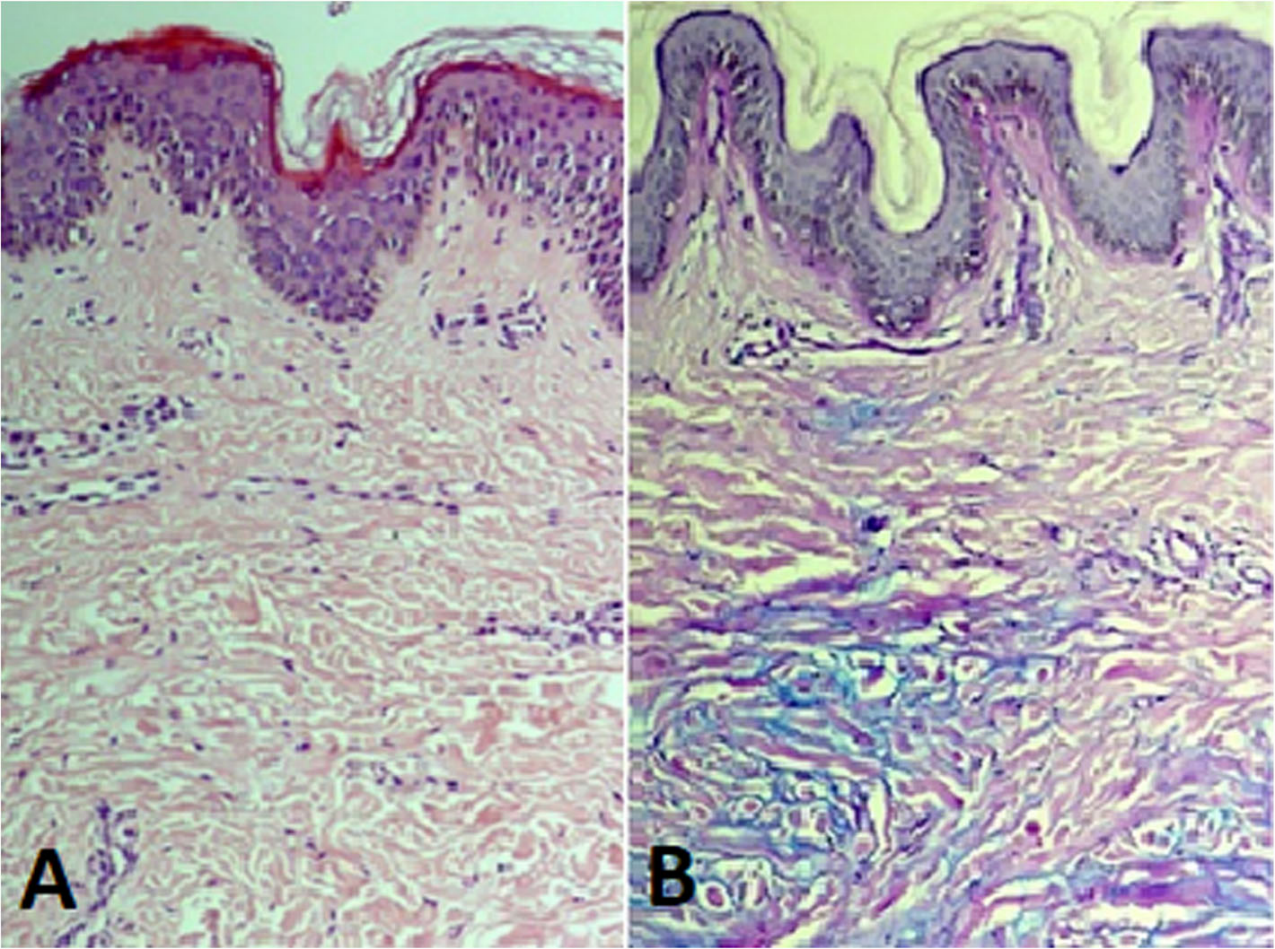
Histopathological findings. Microphotograph: **A** H&E enl. 10x. Skin biopsy shows slight epidermal hyperplasia. No apparent alteration of the dermis. **B** the same specimen stained with Alcian-PAS, which highlights the presence of mucin (light blue) between collagen strands. A-P, enl 10x

We describe a case of a healthy 5-year-old Caucasian boy who presented acute left knee and left elbow swelling accompanied by pain. He denied trauma or other symptoms; 2 weeks earlier, however, he had suffered an upper respiratory tract infection without fever. Laboratory tests (complete blood cell count, hepatorenal function, serum electrolytes, *C-reactive protein* (CRP) and erythrocyte sedimentation rate (ESR), autoantibodies ANA, ENA, anti-dsDNA, mycoplasma serology) were within normal limits and ultrasound examination of the affected joints revealed mild joint effusion. The child was treated with nonsteroidal anti-inflammatory drugs (NSDAIs) for 10 days with clinical resolution. One year later, he presented swelling of the left knee which had been ongoing for 2 months, persisting despite NSDAIs treatment. Ultrasound examination showed the presence of a Baker’s cyst and joint effusion in the suprapatellar recess. Juvenile Idiopathic Arthritis (JIA) was diagnosed and intra-articular steroid injection of the knee was performed. Three months later, the child was admitted to our hospital owing to the persistence of the knee swelling. Ultrasound and Magnetic Resonance Imaging (MRI) of the affected joint (Fig. 1) confirmed the presence of a voluminous Baker’s cyst, but showed the resolution of the joint effusion in the suprapatellar recess. Laboratory tests, including autoimmunity, were negative. Physical examination revealed the presence of some skin lesions: non-tender mobile skin-colored papules localized in the gluteal and umbilical regions, plaques in the right lumbar region, nodular lesions in the left lumbar, left scapular, left anterior and medial femoral regions (Fig. 2). The child’s parents reported that the first skin lesions had appeared 2 years earlier, when connective tissue nevus had been diagnosed. Owing to the increase in size and number of the lesions a skin biopsy was performed: histopathological examination revealed the presence of a perivascular chronic inflammatory infiltrate in the superficial dermis and interstitial mucine deposition in the reticular dermis (Fig. 3). The clinical features and histopathological findings allowed us to diagnose CMI.

Currently our patient has been on follow-up for the last 3 years. Cutaneous manifestations remained almost stable during this period, however he experienced sporadic episodes of knee swelling and pain which resolved with *NSAIDs*.

## Discussion and conclusions

CMI is a persistent dermatosis characterized by the presence of asymptomatic and variably sized skin-colored to erythematous papules, plaques or single nodules, generally localized in the upper extremities and trunk, with benign prognosis [4, 5]. Our case report is particularly interesting because it is the first in which joint involvement has been reported in association with CMI, a disorder that has so far been described as limited to skin involvement. Of note, joint symptoms have been described in other forms of primary cutaneous mucinosis, such as SHJCM, in association with other systemic symptoms like fever, myositis and lymphocytosis [6, 7]. The relationship of arthritis to CMI is not yet clear. Some interesting in vitro studies have revealed immunologic functional characteristics of specific glycosaminoglycans (GAGs): low molecular weight (LMW) hyaluronan (HA) activates macrophages and dendritic cells and plays a role in immune cell recruitment to sites of inflammation [8]; chondroitin sulphate (CS), on the other hand, promotes neutrophil activation, and chondroitin 4-sulphate (C4S) form of CS activates monocytes to produce monokines [9]. HA and CS are increased in dermatomyositis and lupus skin lesions [10]. It has been suggested that in rheumatic diseases the mucinosis could be related to circulating antibodies stimulating the synthesis of glycosaminoglycans by skin fibroblasts. Based on their known immunomodulatory effects, a potential role of these GAGs species in the pathophysiology of these inflammatory skin conditions has been proposed [10]. McAdam et al. reported a case of a 58-year-old Caucasian woman with papular mucinosis who developed severe proximal myopathy, seronegative inflammatory polyarthritis, and marked eosinophilia, suggesting that popular mucinosis may include serious rheumatic manifestations, in addition to skin involvement [11]. The woman’s non-erosive inflammatory polyarthritis, involving both large and small joints, developed 2 years after the onset of skin involvement and was successfully treated with corticosteroid and methotrexate. Synovial histology revealed inflammatory synovitis and, quite unexpectedly, no mucopolysaccharide deposition was found. The unexpected absence of mucopolysaccharide deposition in the synovial membrane makes the hypothesis of a potential role of mucin in the development of inflammatory arthritis less plausible. The result of this study could also raise the question arthritis and CMI: a casual association or a pathogenic correlation? Based on the current knowledge the pathogenic link between skin manifestations and joint involvement in primary cutaneous mucinosis remains to be clarified and therefore we can not exclude that the association described in our patient is accidental. Further histological studies are needed in order to elucidate the pathogenesis of joint involvement in primary mucinosis.

In conclusion, CMI is a rare condition and few cases have been described. Further studies will be necessary in order to better define the clinical findings of different forms of cutaneous mucinosis in children. Owing to the absence of prognostic markers and specific treatment, long-term follow-up and multidisciplinary approach are currently recommendable [12].

## Data Availability

Data sharing is not applicable to this article as no datasets were generated or analysed.

## Abbreviations

GAGs: Glycosaminoglycans
CMI: Cutaneous Mucinosis of Infancy
SHJCM: Self-healing Juvenile Cutaneous Mucinosis
CRP: C-reactive protein
ESR: Erythrocyte Sedimentation Rate
ANA: Antinuclear Antibody
ENA: Extractable Nuclear Antigen
NSAIDs: nonsteroidal anti-inflammatory drugs
JIA: Juvenile Idiopathic Arthritis
MRI: Magnetic Resonance Imaging
LMW: Low molecular weight
HA: Hyaluronan
CS: Chondroitin sulphate
C4S: Chondroitin 4-sulphate
